# Experience-dependent mushroom body plasticity in butterflies: consequences of search complexity and host range

**DOI:** 10.1098/rspb.2017.1594

**Published:** 2017-11-01

**Authors:** Laura J. A. van Dijk, Niklas Janz, Alexander Schäpers, Gabriella Gamberale-Stille, Mikael A. Carlsson

**Affiliations:** Department of Zoology, Stockholm University, 106 91 Stockholm, Sweden

**Keywords:** host specialization, mushroom body, Nymphalidae, plasticity, sensory processing

## Abstract

An ovipositing insect experiences many sensory challenges during her search for a suitable host plant. These sensory challenges become exceedingly pronounced when host range increases, as larger varieties of sensory inputs have to be perceived and processed in the brain. Neural capacities can be exceeded upon information overload, inflicting costs on oviposition accuracy. One presumed generalist strategy to diminish information overload is the acquisition of a focused search during its lifetime based on experiences within the current environment, a strategy opposed to a more genetically determined focus expected to be seen in relative specialists. We hypothesized that a broader host range is positively correlated with mushroom body (MB) plasticity, a brain structure related to learning and memory. To test this hypothesis, butterflies with diverging host ranges (*Polygonia c-album*, *Aglais io* and *Aglais urticae*) were subjected to differential environmental complexities for oviposition, after which ontogenetic MB calyx volume differences were compared among species. We found that the relative generalist species exhibited remarkable plasticity in ontogenetic MB volumes; MB growth was differentially stimulated based on the complexity of the experienced environment. For relative specialists, MB volume was more canalized. All in all, this study strongly suggests an impact of host range on brain plasticity in Nymphalid butterflies.

## 1. Background

The search for a suitable host plant to lay eggs on has proved to be a challenging task for an insect. During host search, the insect may perceive a diversity of sensory information via visual, chemical and mechanosensory cues [1,2], information that is used to select a suitable habitat, patch and ultimately host plant to lay her eggs on [3,4]. The quantity and diversity of sensory information that needs to be processed during host search is very much dependent on the host range of the insect. A larger repertoire of host plants means more potential cues to screen for and discriminate between; as a result, neural capacities will be exceedingly challenged [5–7]. The information processing hypothesis predicts that a generalist consequently has less neural potential left to make quality discrimination between conspecific hosts, leading to lower accuracy [8], longer decision times [9], or both [10,11]. More specialized insects, on the other hand, can focus their neural capacities on only one or a few host-plant species, rendering them more efficient in host plant identification and quality discrimination.

One presumed generalist strategy to improve searching efficiency is to acquire a searching focus during its lifetime, having a filtering effect on sensory inputs and thus reducing information overload [6,12]. Searching attention towards a specific set of host-plant cues is based on the memorized sensory experiences of the ovipositing insect within its current environment [13]. The mushroom body (MB) is an insect brain structure involved in higher-order processing of sensory inputs, including sensory integration, learning and memory [14,15]. Volumetric and architectural changes in the MB and its sub-compartments in response to sensory experiences have been demonstrated repeatedly, especially in social Hymenopteran insects [16], but also some Lepidoptera appear to bear a high potential for ontogenetic plasticity of the MB calyces [17,18]. Studies integrating behavioural and neurological observations have demonstrated a direct link between insect learning and MB expansion in both Hymenopteran [19,20] and Lepidopteran [18] species.

Following from the above, one should expect relative specialist species to rely mostly on their innate searching focus (instinctive behaviour), whereas relative generalists increasingly rely on acquired focus (learned behaviour). Differences in search focus related to host range are most probably occurring at a higher-order processing level, as chemosensory input from host and non-host plants is represented in a similar manner among generalist and specialist butterfly species in the primary olfactory processing centre, the antennal lobe [21].

However, learning possibilities for specialists are not excluded. Even when specialized on only a single host-plant species, experience can still be used to modify preferences for intraspecific plant differences, such as plant sizes, phenological stage [22], or leaf shape [23]. Still, all else being equal, one should expect relative generalists to be more reliant on learning compared with relative specialists, given the bias of scientific evidence towards this hypothesis.

In this study, we used three closely related butterfly species with varying degrees of specialization from the family Nymphalidae, in order to investigate differences in neural plasticity related to search complexity and host range. Included relative specialist species were *Aglais io* and *Aglais urticae*, which were compared with the relative generalist *Polygonia c-album*. For the latter species, two distinct populations (English and Swedish) were investigated with diverging degrees of specialization, allowing intraspecific comparisons.

Ontogenetic divergence of MB volumes induced by environmental complexity was used as a quantitative measure for neural plasticity: MB calyx volume was compared between butterflies that experienced oviposition in environments of varying complexity. We hypothesized that the relative generalists would show higher MB plasticity compared with specialists, because theory predicts that: (i) generalists need to process larger varieties of sensory information owing to their broad host range, and (ii) generalists rely on experience in order to modify host-plant preferences during lifetime.

## 2. Material and methods

### (a) Study system

The terms specialist and generalist are difficult to define and should be used in a relative sense [24,25], meaning that one can only speak of a generalist or specialist species in comparison with another. In this paper, this definition will be adopted when referring to generalist and specialist species.

The study system comprised three butterfly species from the family Nymphalidae: two relative specialist butterfly species (*Ag. io* and *Ag. urticae*) and one relative generalist species (*P. c-album*). From the more generalized species, two divergent populations were studied (English and Swedish) with different degrees of specialization [26]. This study system allowed detection of both inter- and intraspecific effects of specialization on MB plasticity.

*Aglais urticae* and *Ag. io* are both clutch-laying specialists on the stinging nettle *Urtica dioica* (Urticaceae) [27]. *Polygonia c-album* is a generalist species that lays eggs singly. Previous studies have shown that individuals derived from the English population of *P. c-album* are relatively more specialized compared with their Swedish conspecifics [8,26,28–31]. The genetic basis underlying this intraspecific divergence in specialization has also been identified [32]. The host-plant repertoire of Swedish *P. c-album* includes plants from four orders, namely Rosales, Betulales, Salicales and Urticales [33], but the preferred host is *U. dioica* [26]. English *P. c-album* is specialized on *U. dioica* to a relatively high degree [26].

Both host-plant and non-host-plant species were used for oviposition experiments. Included host-plant species of *P. c-album* comprised one herb (*U. dioica*), one shrub (*Ribes alpinum*) and two trees (*Salix caprea* and *Betula pubescens*). Non-host plants for all butterfly species included four herbs (*Alliaria petiolata*, *Lamium album*, *Aegopodium podagraria* and *Cirsium arvense*) and one tree (*Betula pendula*). Plant material was collected around the Stockholm University campus.

### (b) Collection and rearing

*Aglais urticae* and Swedish *P. c-album* individuals were caught in the Stockholm area in May 2016 and bred for one to two generations in the laboratory before being used in the experiments. The eggs were kept in Petri dishes with moistened paper and *U. dioica* leaves at 17°C with a photoperiod of 12 L : 12 D. Hatching larvae were collected and relocated to a larger jar with multiple *U. diocia* stalks, which were constantly watered and kept fresh. Larvae were reared under the same climate regime up until moulting to the third instar. Then, larvae were transferred to 23°C with a photoperiod of 22 L : 2 D, to induce direct development [34]. Jars now contained a maximum of six individuals.

Eggs of English *P. c-album* were obtained from a commercial butterfly provider (Worldwide Butterflies, UK). These eggs were derived from wild-caught *P. c-album* originating from the eastern UK (Norfolk). The eggs and larvae were kept under the same conditions as *Ag. urticae* and Swedish *P. c-album*, but were already relocated to 23°C with a photoperiod of 22 L : 2 D upon moulting to the second instar to speed up development. As English *P. c-album* has a different threshold for direct development, this method still promotes direct developing morphs [34].

Larval clutches of *Ag. io* were collected in June 2016 at their fourth instar at the Stockholm University area. Larvae were reared in large jars with a maximum of six individuals per jar, having constant access to fresh *U. diocia* stalks. The rearing temperature was 23°C with a photoperiod of 22 L : 2 D.

After pupation, all pupae were sexed based on microscopic analysis of the genitals, and kept at 23°C with a photoperiod of 22 L : 2 D until eclosion.

### (c) Treatments

**Figure 1.**
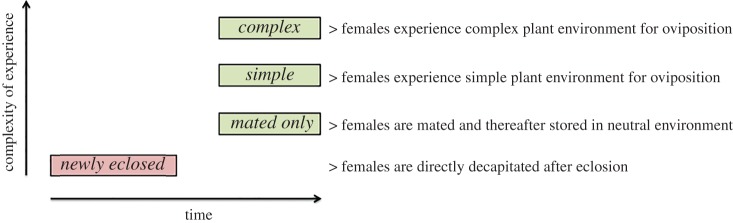
Schematic of the four treatments used; each treatment is presented with its corresponding label. The *x*-axis displays time differences between treatments, and the *y*-axis represents the complexity of the experience for each treatment. (Online version in colour.)

The experiment consisted of four treatments, and for each treatment, at least 10 female individuals per species (or population) were included (figure 1). The treatments were initiated as soon as possible after eclosion of the pupae, with a maximum time of 12 h in between.

#### (i) Control treatments

Females included in the *newly eclosed* treatment were decapitated directly after eclosion. Females used in all remaining treatments were mated; butterflies were moved to mating cages (70 × 70 × 50 cm) with a daylight regime of 7 L : 17 D. Each cage contained a maximum of 20 butterflies with approximately equal numbers of males and females. The experimental treatments using the mated females were initiated as soon as mating was finished. For the *mated only* treatment, mated females were stored in the neutral environment (climate chamber of 17°C, 8 L : 16 D) for four subsequent days (96 h). For all treatments, the mating status of the mated females was confirmed at the end of the experiment via dissection of the abdomen to check for the presence of a spermatophore.

#### (ii) Plant-environment treatments

**Figure 2.**
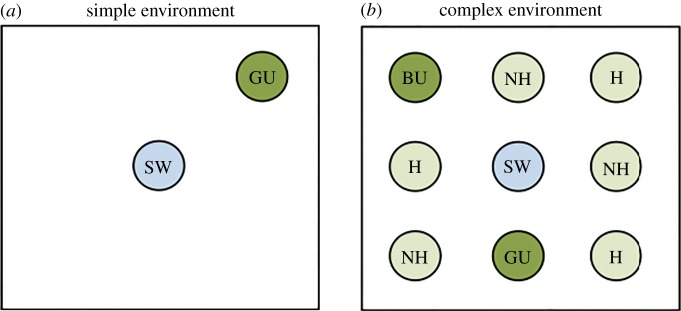
Example set-up for a simple (*a*) and complex (*b*) environment. SW, sugar water; GU, good quality *U. dioica*; BU, bad quality *U. dioica*; H, other host plants (of *P. c-album*); NH, non-host plants. (Online version in colour.)

Females in the *simple* or *complex* treatment were transferred to a simple or a complex plant environment, respectively, and stayed in this environment for four subsequent days (96 h). The cages of both the simple and complex environments (70 × 70 × 50 cm) were kept at 27°C during daytime and illuminated by a light bulb (Solar Raptor HID 50 W), with a daylight regime of 8 L : 16 D. All plants used in the complex and simple environments were presented in glass bottles filled to the top with water. Notably, all conditions were similar in both the simple and complex environments, apart from the plant species presented to the ovipositing butterflies. The simple environment consisted of one *U. dioica* of good quality and a tower with a sponge of sugar water (diluted approximately to 1 : 4) (figure 2*a*). The position of *U. dioica* in the cage was randomly changed each day to prevent position effects. The complex environment consisted of eight plants, both host and non-host plants, and a tower with a sponge of sugar water (1 : 4). For an example of the set-up of the complex environment, see figure 2*b*. The positions of all plants in the cage were changed each day to prevent position effects. The identity of the non-host plants also differed between days in order to increase environmental complexity. Plants present at all times in the cages were *U. dioica* (both good and bad quality), *R. alpinum*, *S. caprea* and *B. pubescens*; these plants are all included in the host-plant repertoire of *P. c-album*. The plants *Al. petiolata*, *B. pendula*, *L. album*, *Ae. podagraria* and *C. arvense* were only temporarily present in the complex environment (1–2 days). These plants are non-host plants for all used butterfly species and naturally grow interspersed in the host-plant habitat; these plants were included to make the environment more complex and to potentially confuse the ovipositing butterfly. For an overview of the combinations of plant species used in the complex environment, see the electronic supplementary material, appendix S1. Females were subjected to the environments mostly individually, but sometimes in pairs of two because of space restrictions. We did not expect singular versus pairwise occurrence of individuals to have an effect on treatment outcome, and hence, all individuals were treated as being independent.

#### (iii) Age of females in the treatments

The average age of females among treatments was kept constant as much as possible within a species, to diminish age-related variation in neuropil volumes. Age fluctuations were introduced during the mating process, as the time before getting mated varied between individuals. For all species except *Ag. urticae*, similar average ages among treatments were successfully maintained (see the electronic supplementary material, appendix S2). For *Ag. urticae*, the duration of the mating process was highly variable among individuals; for some females, it took up to a few weeks before getting mated. This inevitably caused age divergence among treatments, with the lowest average age in the *mated only* (15 days) and the highest in the *simple* and *complex* treatment (21 days; electronic supplementary material, appendix S2 and table S1). Note that even though time between eclosion and mating varied per individual, the time spent in the different environments was kept constant at all times.

### (d) Immunohistochemistry and brain scanning

Females were decapitated directly after completion of a treatment. From the decapitated heads, the mouthparts were removed and small holes were pierced in the compound eyes, which was done in phosphate buffer (PB) 0.1 M. The heads were fixed overnight in 4% paraformaldehyde (diluted in PB 0.1 M, pH 7.4). After fixation, heads were washed in PB 0.1 M and phosphate-buffered saline with 2.5% Triton-X (PBS-tx). Brains were dissected in PBS-tx, and thereafter incubated for 72 h with the primary antibody, mouse monoclonal anti-synapsin (anti-SYNORF1; Developmental Studies Hybridoma Bank, Iowa City, IA, USA), diluted 1 : 20 with PBS-tx and 0.5% bovine serum albumin (BSA). Brains were washed with PBS-tx, and then incubated for 48 h with the secondary antibody, anti-mouse Alexa 546 (Invitrogen, Stockholm, Sweden), diluted 1 : 500 with PBS-tx and 0.5% BSA. After staining, the brains were carefully washed first in PBS-tx and then PBS, and subsequently stored at 4°C in PBS with 0.02% sodium azide. Finally, all brains were dehydrated in a series of increasing alcohol concentrations (30–100%), and cleared and stored in methyl salicylate at 4°C.

### (e) Brain imaging and analyses

Brains were mounted for scanning in methyl salicylate with 500 µm spacers. Brains were scanned as whole mounts with a Zeiss LSM 780 META (Zeiss, Jena, Germany) confocal laser scanning microscope, and images were obtained with a 10× air objective (Plan-Apochromat 10×/0.45 M27) at a 1024 × 1024 pixel resolution. Laserline DPSS 561 nm was used for excitation with a pinhole size of 1 airy unit. A digital zoom of 1.1 for *Ag. io* and 1.2 for the other species was applied. Each brain was scanned with about 100 sections, with a step size of 3 µm. The ZEN black 2011 software was used for image acquisition.

Three-dimensional surface reconstructions of confocal stacks were generated using the segmentation tool in the AMIRA 4.1 software (Mercury Computer Systems GmbH, Berlin, Germany), thereby producing a detailed image of the central brain. All synapsin immunoreactive regions of the brain, excluding the optic lobes, were included in the reconstruction. From these reconstructions, volume measurements were performed for each brain structure of interest (central brain, antennal lobes and MB calyces).

### (f) Statistical analyses

Statistical analyses were performed in R v. 3.2.1 [35]. Volume measurements (µm^3^) of the central brain as a whole, antennal lobe and MBs were used for volumetric comparisons of brain tissues. To correct for the effect of the size of the central brain on other brain structures, relative MB calyx (rCA) volume (see equation (2.1)) and relative antennal lobe (rAL) volume (see equation (2.2)) were calculated:2.1
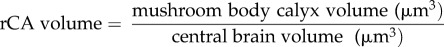
and2.2
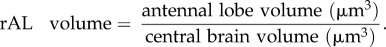


All volume measurements were tested for normal distribution using the Anderson Darling test in combination with visual inspection of model residuals. After normality was confirmed, the effects of treatments, species and treatment × species on brain tissue volume differences were analysed with a two-way ANOVA and post hoc Tukey test. Dispersion of samples within treatments was calculated using the standard error. Linear regression analyses were performed to identify potential correlations between brain structure volumes among treatments and species.

## 3. Results

### (a) Brain reconstructions

**Figure 3.**
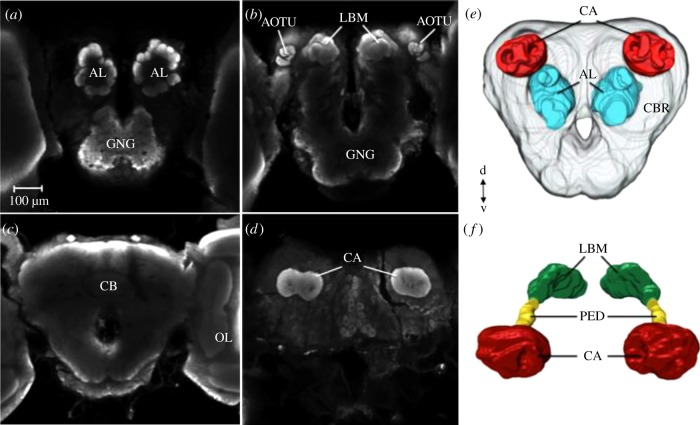
Anatomy of the butterfly central brain. (*a*–*d*) Anti-synapsin immunofluorescence in single confocal sections of the central brain of *Ag. urticae*, depicted from four focal depths, anterior to posterior, showing (*a*) antennal lobes (AL) and gnathal ganglia (GNG), (*b*) lobes mass of the MB (LBM), anterior optic tubercle (AOTU) and GNG, (*c*) central body (CB) and part of the optic lobes (OL) and (*d*) MB calyces (CA). (*e*) Surface reconstruction of the butterfly central brain, with MB calyces (CA, red), antennal lobes (AL, blue) and the central brain (CBR, grey). (*f*) Surface reconstruction of the mushroom bodies. CA, calyx; PED, peduncle; LBM, lobes mass of the MB. Only the calyces were used for further analyses on the MB volume. Scale bar, 100 µm in (*a*–*d*).

The antennal lobes, MB calyces and the central brain were reconstructed for all treated females (*Ag. io*, *n* = 38; *Ag. urticae*, *n* = 28; English *P. c-album*, *n* = 35 and Swedish *P. c-album*, *n* = 36). The butterfly brain anatomy is depicted in figure 3*a*–*d*, including an example of a brain reconstruction (figure 3*e*) and a reconstruction of the complete MB (figure 3*f*).

### (b) Central brain volumes

Central brain volumes varied among species (ANOVA: *F*_3,121_ = 56.7, *p* < 0.0001), but showed only minor ontogenetic fluctuations within species (see the electronic supplementary material, appendix S3). *Aglais io* exhibited significantly larger central brain volumes compared with *Ag. urticae* and *P. c-album* (*p* < 0.0001), whereas the central brain sizes of the latter two species were not significantly different from each other. This difference was already apparent directly after eclosion, indicating congenital differences in central brain size (ANOVA, *F*_9,121_ = 1.7; followed by post hoc Tukey, *Ag. urticae* and Swedish *P. c-album*, *p* < 0.0001; English *P. c-album*, *p* = 0.003). Ontogenetic growth of the central brain was only observed upon ageing (from the *newly eclosed* treatment to aged treatments) (ANOVA, *F*_9,121_ = 1.7). However, significant volume differences among treatments with females of the same age were never observed (*mated only*, *simple* or *complex* treatment).

### (c) Relative antennal lobe volumes

Antennal lobe volume was measured as a control, as input to the MB is mainly relayed from this structure. The rAL volumes showed minor volumetric differences among treatments, though these differences were never significant (ANOVA: *F*_9,121_ = 2.2) (see the electronic supplementary material, appendix S4). No clear ontogenetic trend could be obtained from the data. When comparing among species (ANOVA, *F*_3,121_ = 56.0), *Ag. io* (*n* = 38) and *Ag. urticae* (*n* = 28) had significantly smaller rAL volumes compared with *P. c-album* (*n* = 71; *p* < 0.0001). This significant volumetric difference was already present when comparing *newly eclosed* butterflies, indicating that rAL volumes differ congenitally among generalist and specialist species (ANOVA, *F*_9,121_ = 2.16; post hoc Tukey, Swedish *P. c-album* versus *Ag. io* and *Ag. urticae*, *p* < 0.0001; English *P. c-album* versus *Ag. io* and *Ag. urticae*, *p* = 0.026 and 0.001, respectively). Linear regression revealed that this interspecific difference of rAL volume positively correlated with rCA volume (*p* < 0.0001, *R*^2^ = 0.26; see the electronic supplementary material, appendix S6). *Polygonia c-album* exhibited the largest rAL volumes in combination with the largest rCA volumes; for both *Aglais* species, these brain structures were smaller. Note that this correlation was fuelled only by interspecific differences rather than differences among treatments—no significant positive correlation could be obtained from intraspecific comparisons.

### (d) Relative mushroom body volumes

**Figure 4.**
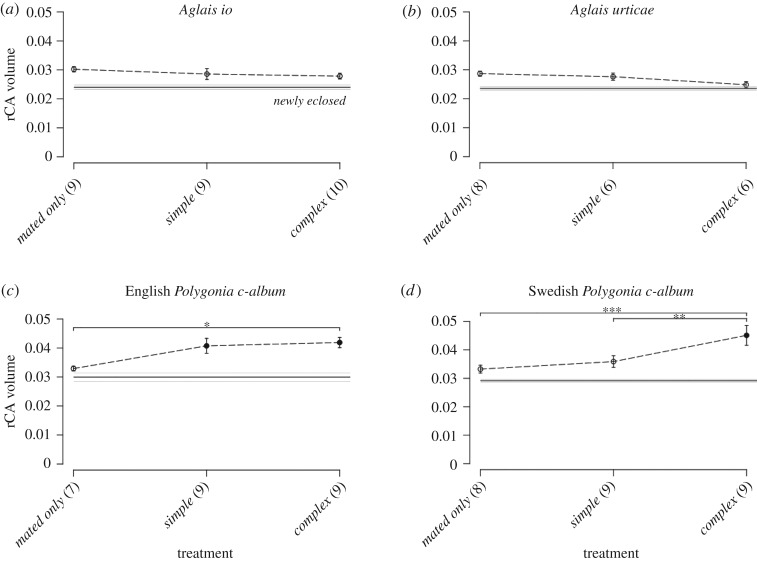
The rCA volume of all species for all treatments: (*a*) *Ag. io*, (*b*) *Ag. urticae*, (*c*) English *P. c-album* and (*d*) Swedish *P. c-album*. The *newly eclosed* treatment is depicted as a horizontal baseline, with grey lines on either side representing the standard error. Filled points are significantly larger than the *newly eclosed* treatment, but open points are not. Note that the lines between treatment points do *not* indicate timeseries, but rather visualize the reaction norm of the connected treatments (ANOVA and post hoc Tukey's test; *p < 0.05, **p < 0.01, ***p < 0.001).

Relative volumes of MB calyces among treatments are depicted for all species in figure 4*a*–*d*. When comparing the overall pattern of rCA volume among species, it is evident that both English and Swedish *P. c-album* bear more ontogenetic plasticity in rCA volume compared with the *Aglais* species. Significant differences of rCA volume between species were identified; the relative specialist species (*Ag. urticae* and *Ag. io*) showed a significant difference in rCA size compared with the relative generalists (English and Swedish *P. c-album*) (ANOVA: *F*_3,121_ = 40.3, *p* = <0.0001). Furthermore, a significant interaction between species and treatment was present (ANOVA: *F*_9,121_ = 4.9, *p* < 0.0001). Below, the observed trends among treatments are given for each species.

#### (i) *Aglais io* and *Aglais urticae*

For both species, females from the *mated only* (*Ag. io* and *Ag. urticae*; *n* = 9 and 8, respectively) did not show significant rCA volume increase compared with *newly eclosed* (*n* = 10 and *n* = 8; post hoc Tukey's test, *p* = 0.28 and *p* = 0.75, respectively) (figure 4*a*,*b*). Also when subjected to plant experience (*simple* and *complex* treatment), *Ag. io* and *Ag. urticae* lacked significant rCA expansion for both the *simple* (*n* = 9 and *n* = 6; *p* = 0.99 and *p* = 1, respectively) and *complex* (*n* = 10 and *n* = 6; *p* = 0.99 and *p* = 0.98, respectively) treatments compared with *mated only*. Furthermore, small standard errors indicate that variation between individuals within treatments was relatively low (despite the relatively broad age range of *Ag. urticae* females).

#### (ii) Polygonia c-album

Females from the *mated only* treatment for both Swedish and English individuals (*n* = 8 and 7, respectively) showed comparable rCA volumes to those of females from the *newly eclosed* treatment (*n* = 10 and 9; post hoc Tukey's test, *p* = 0.92 and 0.99, respectively) (figure 4*c*,*d*). Then, in contrast to the *Aglais* species, females of *P. c-album* showed significant rCA volumetric increases upon plant experience. The rCA volume of Swedish *P. c-album* remained similar between females of the *mated only* and *simple* treatments (*n* = 9 and *p* = 0.99), but significantly increased when females were subjected to the complex plant environment (*n* = 9) (*mated only* versus *complex*, *p* = 0.0002; *simple* versus *complex*, *p* = 0.009) (figure 4*d*). English *P. c-album* showed a slightly divergent pattern here; namely, females already showed a tendency towards larger rCA volumes when experiencing the simple environment, though not significantly so (*n* = 9) (*mated only* versus *simple*, *p* = 0.11) (figure 4*c*). The rCA volume then increased significantly when females were subjected to the complex environment (*n* = 9) (*mated only* versus *complex*, *p* = 0.023). Here, no significant difference was present between rCA volumes of females in the *simple* and *complex* treatment (*p* = 1). Individual variation within treatments was large for both English and Swedish *P. c-album*, especially in the *simple* and *complex* treatments, resulting in large standard errors.

## 4. Discussion

The results confirmed our hypothesis and showed that the generalist species *P. c-album* exhibited higher neural plasticity compared with the specialist species *Ag. io* and *Ag. urticae*. Oviposition in a plant environment induced a significant MB calyx volume increase for *P. c-album* females, where rCA volumes increased significantly upon oviposition in a complex environment. This rCA volume expansion upon plant experience was absent for *Ag. urticae* and *Ag. io*; these species showed a more canalized rCA volume among treatments. Overall, the generalist and specialist species showed significantly divergent rCA and rAL volumes among treatments.

Although the results are in accordance with our hypothesis, other explanations cannot be ruled out. For example, the *Polygonia* butterflies are single-egg layers, whereas the *Aglais* species are clutch layers. One could argue that single-egg layers gather more experience compared with clutch layers, as they make a host-plant choice for (almost) each individual egg. On the other hand, the actual number of evaluated plants may not differ quite as much between single-egg-laying and clutch-laying females. As a clutch-laying female has to be more discerning, she arguably has to evaluate and reject many plants for every successful oviposition event.

Significant differences in ontogenetic central brain volumes were observed, but only among treatments with different ages. No significant trends of central brain volume increase or decrease were seen upon plant experience when comparing age-related treatments (electronic supplementary material, appendix S3). This finding suggests that the central brain can grow as a function of age, but does not increase in size upon plant or mating experience. When comparing among species, *Ag. io* exhibited a significantly larger congenital central brain volume compared with the other species, an expected result given the larger body size of *Ag. io*.

The antennal lobes did not show any obvious ontogenetic trend for volume increase or decrease (electronic supplementary material, appendix S4). Rather, congenital differences were identified, revealing that *P. c-album* generally exhibited larger antennal lobes compared with the *Aglais* species. As glomeruli number is a highly conserved trait in the Lepidoptera [21,36,37], these size differences are likely to be attributed to either larger volumes of individual glomeruli or larger volume of the antennal lobe hub owing to increased interglomerular branching [17,38]. The results further showed a significant correlation between rAL and rCA volumes among treatments (electronic supplementary material, appendix S6), where the generalist species exhibited the largest overall volumes of both neuropils. Considering the functional relatedness of these neuropils, correlation is likely to reflect causation.

Regarding MB calyx volumes, the results showed the highest plasticity in rCA volume for both English and Swedish *P. c-album*. Not only did the rCA volumes significantly change among treatments, but they also showed remarkable variation within treatments. This within-treatment variation was, expectedly, strongest for the females that experienced a plant environment. In these environments, individual females can adopt different behaviours, such as varying flight-, plant screening- or egg-laying activities [39,40], causing greater differences in the experiences each female gathers from her environment. Furthermore, *P. c-album* is known for its intraspecific variation in specificity; the degree of specialization varies between and even within populations [41,42]. This too could have contributed to diverging MB volumes in a plant environment, with smaller MB volumes expected for the more specialized individuals. Ontogenetic growth of the MB has been related both to adult neurogenesis of Kenyon cells [43] and enhanced dendritic branching [44].

By contrast, *Ag. io* and *Ag. urticae* showed more canalized MB volumes both among and within treatments. Both specialist species lack experience-based growth of MB volume, in contrast to the generalist species. As CA plasticity has been shown to positively correlate to insect learning [18–20], these findings suggest that *P. c-album* exploited learning during oviposition in the (complex) plant environments, whereas for *Ag. io* and *Ag. urticae* learning is presumably limited or even absent during the experiment. Instead, a genetically fixed searching focus may have been used to efficiently locate their preferred host (*U. dioica*) during oviposition, suggesting that host-plant search for the relative specialists is more based on innate behaviour rather than derived. Innate focus is optimal in a sufficiently predictable environment with reliable host availability, as it contributes to a more time- and energy-efficient host-searching strategy [6,45]. Acquired searching focus, on the other hand, equips the insect with behavioural plasticity, a trait which is especially beneficial when coping with a heterogeneous environment and unpredictable host availability [6].

While both Swedish and English females of *P. c-album* demonstrated experience-based rCA volume expansion in response to the plant treatments, our results indicated a difference between them in how they responded to the simple and complex environments. Interestingly, English *P. c-album* already had a tendency for rCA volume expansion in the simple environment (though not significant), which was not the case for the Swedish. This observation could be explained by the higher degree of specialization of English *P. c-album* on *U. dioica* [26], which is related to the enhanced capacity to differentiate between conspecific qualities of the host plant [8]; an ability that could already be exploited in the simple environment with *U. dioica* only. This finding suggests that learning might be involved in the process of making conspecific quality distinctions. However, it must be noted that individual volumetric differences within treatments were rather large. Hence, while in line with expectations, this conclusion should be treated with some caution.

As English *P. c-album* is more specialized compared with its Swedish conspecifics, one might wonder why this did not coincide with a more canalized pattern of ontogenetic rCA volume for English *P. c-album*. However, considering the relatively recent polyphagous past of English *P. c-album* [24], as well as the fact that they are not strictly specialized, it might be unlikely to assume that selection pressures on MB plasticity are strong enough to cause evolutionary change towards MB canalization within a relatively short evolutionary time.

Although learning provides an animal with behavioural benefits and flexibility, this ability comes at a cost, as neural tissues are energetically expensive to develop and maintain [46]. Furthermore, learning a certain behaviour takes time [45], which may reduce egg-laying efficiency, and it renders the insect susceptible to potential predators for a longer period of time [6]. Regarding the investment in neural tissue, learning entails both ‘induced’ and ‘global costs’ [18]. Global costs will always be accrued by individuals with a genetically high learning capacity, even if learning will not be exploited during their lifetime [18,47]. Induced costs, on the other hand, are only paid in environments where learning is actually needed [18]. This ‘pay as you go’ system promotes the evolutionary conservation of learning by dampening the lifetime costs of neural investments [18,48]. In this study, the demonstrated ontogenetic rCA plasticity of *P. c-album* can be interpreted as an induced cost of learning.

Given the costs, evolution towards high learning capacity should be expected to predominantly occur when a species can behaviourally benefit from it [49]. Generalists are expected to have a behavioural advantage of learning as it enables them to develop a searching focus that is adjusted to their experienced environment [6]. Learning can be seen as an adaptive trait to deal with the costs of being a generalist [6] and, consequently, might be an important mechanism behind the evolutionary conservation of generalism. Also, spatial orientation has been shown to imply CA plasticity; numerous studies have demonstrated CA expansion in foraging honeybees [44,50] and other Hymenoptera [51]. Recently, spatial orientation was also related to CA expansion in *Heliconius* butterflies, in a study by Montgomery *et al*. [17]. *Heliconius* butterflies feed on pollen, which requires sophisticated spatial orientation during foraging; a behavioural trait that is highly likely to put strong selection pressures on neural plasticity during evolution [17]. Neuroplasticity turns out to have profound impacts on ecological and evolutionary processes; our study additionally suggests a role for neuroplasticity in host range evolution of Nymphalid butterflies.

## 5. Conclusion

The results in this study confirm our hypothesis and show a positive correlation between generalism and neural plasticity. The relative generalist species *P. c-album* showed plasticity in ontogenetic MB volumes and expansion of the rCA volume upon plant experience. By contrast, the more specialized species, *Ag. urticae* and *Ag. io*, exhibited canalized ontogenetic MB volumes with no rCA volumetric increase upon plant experience. In addition to this, the generalist species possessed larger overall rCA and rAL volumes compared with the specialist species, a difference that was in line with expectations.

## Supplementary Material

Appendices 1 - 6

## Data accessibility

All relevant data associated with this study are available from the Dryad digital repository, http://dx.doi.org/10.5061/dryad.hr1f1 [52].
