# Supplementary material for: Experience-dependent mushroom body plasticity in butterflies: consequences of search complexity and host range
Source: Proc Biol Sci. 2017 Nov 1;284(1866):20171594. doi: 10.1098/rspb.2017.1594 (PMC5698644; doi:10.1098/rspb.2017.1594)
Supplement: Appendices 1 - 6 [file rspb20171594supp1.pdf]

**Appendix 1**: Plant species combinations in the complex environment

| Complex environment – Day 1 | Complex environment – Day 2 |
|---|---|
| Good quality *Urtica dioica*<br>Bad quality *Urtica dioica*<br>*Ribes alpinum*<br>*Salix caprea*<br>*Betula pubescens*<br><br>*Alliaria petiolata* 2x<br>*Betula pendula* | Good quality *Urtica dioica*<br>Bad quality *Urtica dioica*<br>*Ribes alpinum*<br>*Salix caprea*<br>*Betula pubescens*<br><br>*Betula pendula* 2x<br>*Lamium album* |
| Complex environment – Day 3 | Complex environment – Day 4 |
| Good quality *Urtica dioica*<br>Bad quality *Urtica dioica*<br>*Ribes alpinum*<br>*Salix caprea*<br>*Betula pubescens*<br><br>*Lamium album* 2x<br>*Aegopodium podagraria* | Good quality *Urtica dioica*<br>Bad quality *Urtica dioica*<br>*Ribes alpinum*<br>*Salix caprea*<br>*Betula pubescens*<br><br>*Aegopodium podagraria* 2x<br>*Cirsium arvense* |

**Appendix 2**: Age range, average and standard deviation of all species per treatment

**Table 1**. Age range, average and standard deviation per treatment of *Aglais urticae*

| Treatment | Age range (days) | Average age (days) | Standard deviation |
|---|---|---|---|
| *Mated only* | 7-24 | 15.2 | 5.5 |
| *Simple* | 16-33 | 21.5 | 5.1 |
| *Complex* | 12-29 | 20.4 | 5.1 |

**Table 2**. Age range, average and standard deviation per treatment of *Aglais io*

| Treatment | Age range (days) | Average age (days) | Standard deviation |
|---|---|---|---|
| *Mated only* | 6-13 | 9.0 | 2.0 |
| *Simple* | 8-14 | 10.3 | 2.4 |
| *Complex* | 8-13 | 9.6 | 2.2 |

**Table 3**. Age range, average and standard deviation per treatment of English *Polygonia c-album*

| Treatment | Age range (days) | Average age (days) | Standard deviation |
|---|---|---|---|
| *Mated only* | 6-15 | 9.3 | 2.9 |
| *Simple* | 8-13 | 9.2 | 1.9 |
| *Complex* | 8-12 | 9.3 | 1.5 |

**Table 4**. Age range, average and standard deviation per treatment of Swedish *Polygonia c-album*

| Treatment | Age range (days) | Average age (days) | Standard deviation |
|---|---|---|---|
| *Mated only* | 6-11 | 8.3 | 1.5 |
| *Simple* | 6-11 | 7.9 | 1.4 |
| *Complex* | 6-11 | 8.0 | 1.3 |

**Appendix 3**: Central brain volumes of all species per treatment

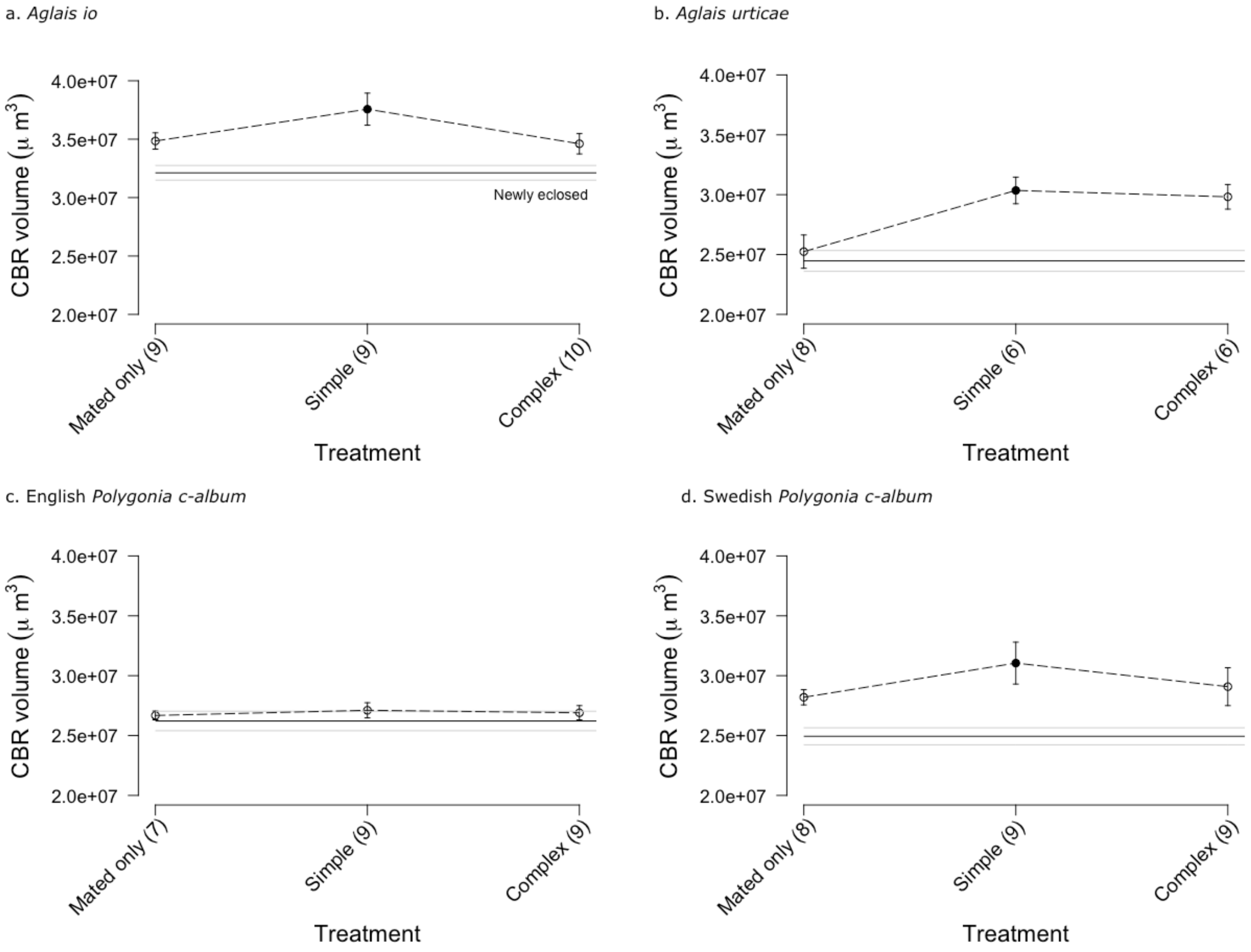


The central brain (CBR) volume in $\mu m^3$ of all species for all treatments: a) *A. io*, b) *A. urticae*, c) English *P. c-album*, d) Swedish *P. c-album*. Treatment *Newly eclosed* is depicted as a horizontal baseline, with grey lines on either sides representing the standard error. Filled points are significantly larger than the *Newly eclosed* treatment, and open points are not. Note that the lines between treatment-points do *not* indicate time series, but rather visualize the reaction norm of the connected treatments. (ANOVA and post hoc Tukey test).

**Appendix 4**: Antennal lobe volumes of all species per treatment

a. *Aglais io*

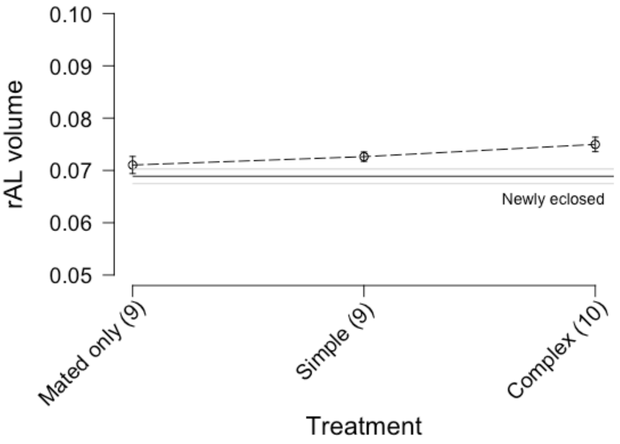


b. *Aglais urticae*

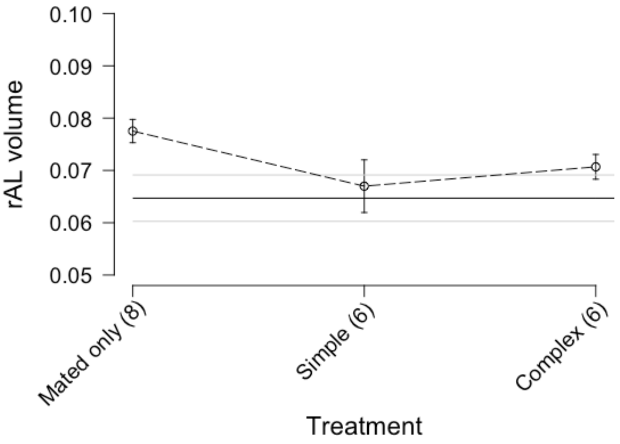


c. English *Polygonia c-album*

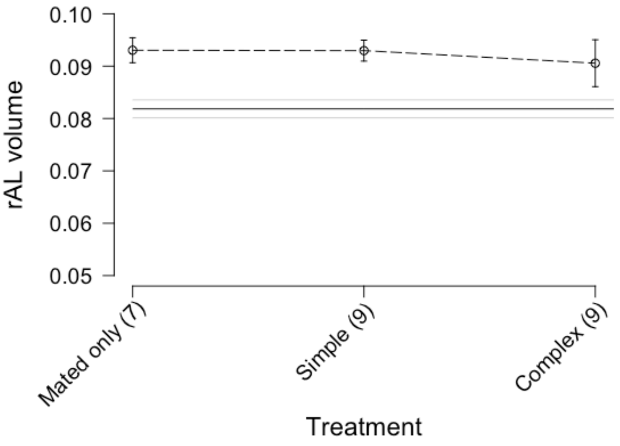


d. Swedish *Polygonia c-album*

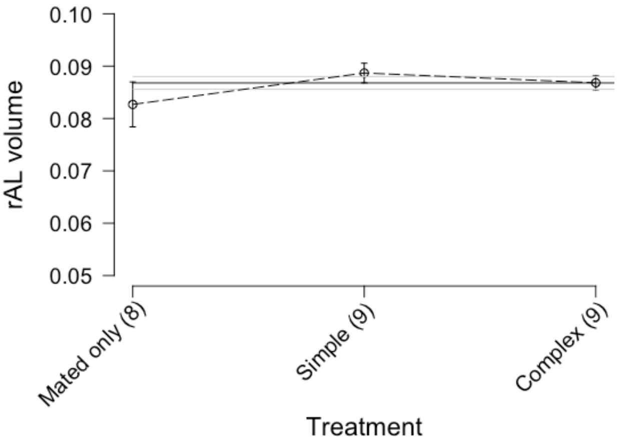


The relative antennal lobe (rAL) volume in $\mu m^3$ of all species for all treatments: a) *A. io*, b) *A. urticae*, c) English *P. c-album*, d) Swedish *P. c-album*. Treatment *Newly eclosed* is depicted as a horizontal baseline, with grey lines on either sides representing the standard error. Filled points are significantly larger than the *Newly eclosed* treatment, and open points are not. Note that the lines between treatment-points do *not* indicate time series, but rather visualize the reaction norm of the connected treatments. (ANOVA and post hoc Tukey test).

**Appendix 5**: Absolute mushroom body calyx volumes of all species per treatment

a. *Aglais io*

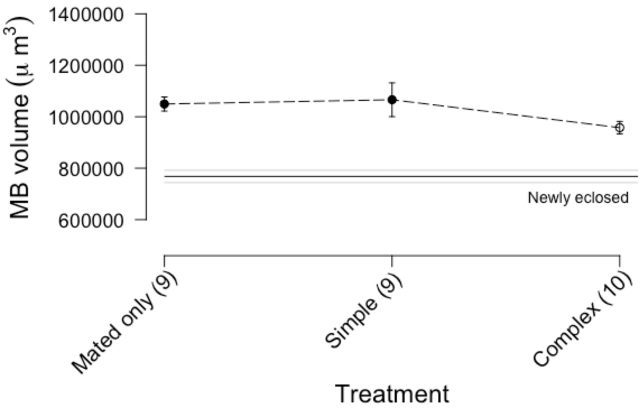


b. *Aglais urticae*

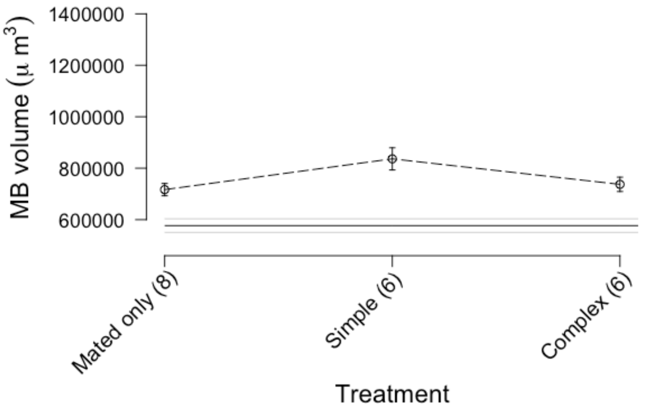


c. English *Polygonia c-album*

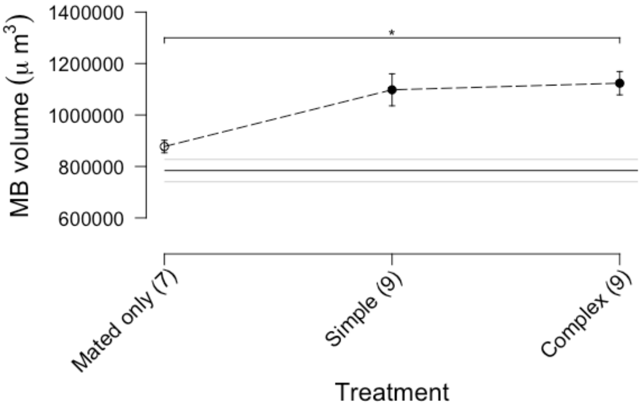


d. Swedish *Polygonia c-album*

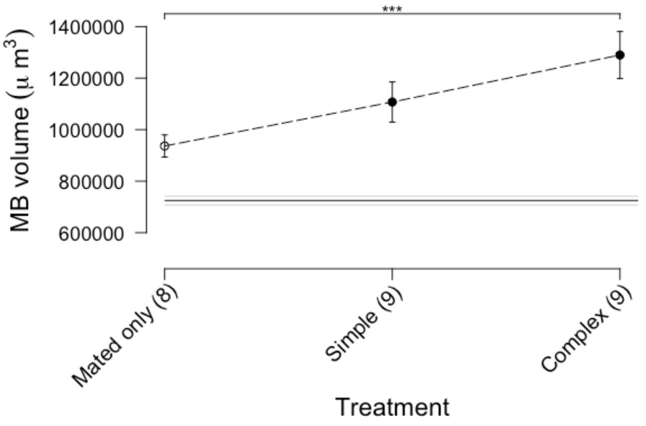


The absolute mushroom body calyx volume in $\mu m^3$ of all species for all treatments: a) *A. io*, b) *A. urticae*, c) English *P. c-album*, d) Swedish *P. c-album*. Treatment *Newly eclosed* is depicted as a horizontal baseline, with grey lines on either sides representing the standard error. Filled points are significantly larger than the *Newly eclosed* treatment, and open points are not. Note that the lines between treatment-points do *not* indicate time series, but rather visualize the reaction norm of the connected treatments. (ANOVA and post hoc Tukey test; * $p < 0.05$, *** $p < 0.001$).

**Appendix 6**: Linear regression analysis of rMB volume against rAL volume

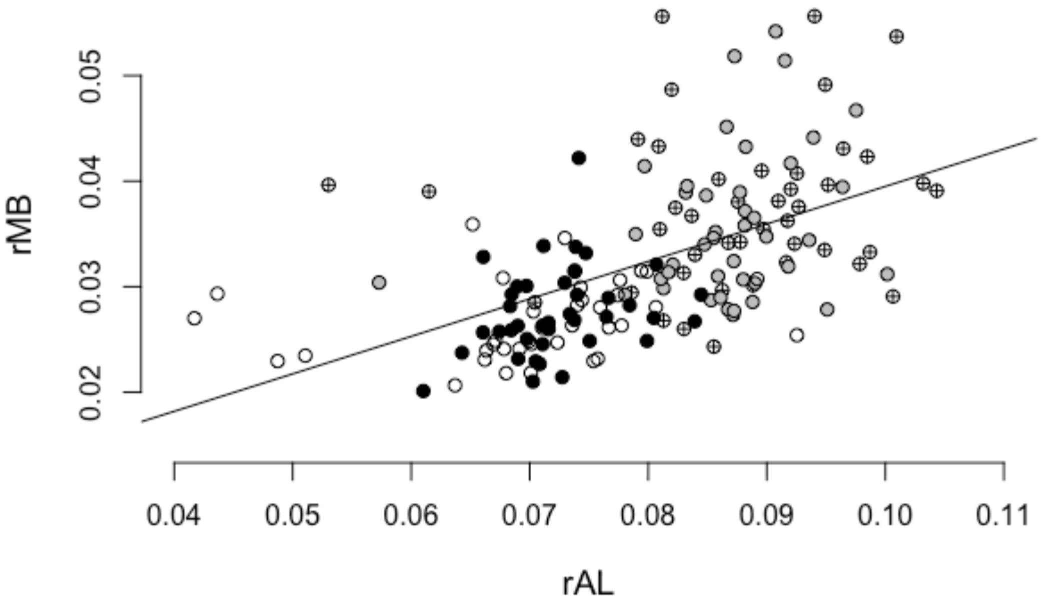


Linear regression analysis of relative mushroom body calyx (rMB) volume in $\mu m^3$ against relative antennal lobe (rAL) volume for all species, all treatments combined. Open circles = *A. urticae*, filled black circles = *A. io*, filled grey circles = Swedish *P. c-album*, crossed circles = English *P. c-album*, $p < 0.0001$, $R^2 = 0.26$.
